# Identification and characterization of a nontypeable *Haemophilus influenzae *putative toxin-antitoxin locus

**DOI:** 10.1186/1471-2180-4-30

**Published:** 2004-07-26

**Authors:** Dayle A Daines, Justin Jarisch, Arnold L Smith

**Affiliations:** 1 Nonproliferation, Arms Control, and International Security Directorate, Lawrence Livermore National Laboratory, L-501, 7000 East Avenue, Livermore CA 94550-9698, USA; 2 Bacterial Pathogenesis Program, Seattle Biomedical Research Institute, 307 Westlake Avenue North, Suite 500, Seattle, WA 98109-5219, USA

## Abstract

**Background:**

Certain strains of an obligate parasite of the human upper respiratory tract, nontypeable *Haemophilus influenzae *(NTHi), can cause invasive diseases such as septicemia and meningitis, as well as chronic mucosal infections such as otitis media. To do this, the organism must invade and survive within both epithelial and endothelial cells. We have identified a facilitator of NTHi survival inside human cells, virulence-associated protein D (*vapD*_*Hi*_, encoded by gene HI0450). Both *vapD*_*Hi *_and a flanking gene, HI0451, exhibit the genetic and physical characteristics of a toxin/antitoxin (TA) locus, with VapD_*Hi *_serving as the toxin moiety and HI0451 as the antitoxin. We propose the name VapX_*Hi *_for the HI0451 antitoxin protein. Originally identified on plasmids, TA loci have been found on the chromosomes of a number of bacterial pathogens, and have been implicated in the control of translation during stressful conditions. Translation arrest would enhance survival within human cells and facilitate persistent or chronic mucosal infections.

**Results:**

Isogenic mutants in *vapD*_*Hi *_were attenuated for survival inside human respiratory epithelial cells (NCI-H292) and human brain microvascular endothelial cells (HBMEC), the *in vitro *models of mucosal infection and the blood-brain barrier, respectively. Transcomplementation with a *vapD*_*Hi *_allele restored wild-type NTHi survival within both cell lines. A PCR survey of 59 *H. influenzae *strains isolated from various anatomical sites determined the presence of a *vapD*_*Hi*_allele in 100% of strains. Two isoforms of the gene were identified in this population; one that was 91 residues in length, and another that was truncated to 45 amino acids due to an in-frame deletion. The truncated allele failed to transcomplement the NTHi *vapD*_*Hi *_survival defect in HBMEC. Subunits of full-length VapD_*Hi *_homodimerized, but subunits of the truncated protein did not. However, truncated protein subunits did interact with full-length subunits, and this interaction resulted in a dominant-negative phenotype. Although *Escherichia coli *does not contain a homologue of either *vapD*_*Hi *_or *vapX*_*Hi*_, overexpression of the VapD_*Hi *_toxin *in trans *resulted in *E. coli *cell growth arrest. This arrest could be rescued by providing the VapX_*Hi *_antitoxin on a compatible plasmid.

**Conclusion:**

We conclude that *vapD*_*Hi *_and *vapX*_*Hi *_may constitute a *H. influenzae *TA locus that functions to enhance NTHi survival within human epithelial and endothelial cells.

## Background

Culturable *Haemophilus influenzae *are acquired in the nasopharynx shortly after birth, and are thought to persist throughout life. *H. influenzae *adheres to and penetrates into and between cultured human respiratory epithelial cells, a mechanism that may contribute to its persistence in chronic obstructive pulmonary disease (COPD) and cystic fibrosis (CF) patients [1,2]. *H. influenzae *can be found in the respiratory tracts of these patients even after they have undergone antibiotic treatment [3]. As well, COPD sputum cultures can be sterile, while *H. influenzae *can still be isolated from the subepithelial matrix [4]. Finally, we have found in a recent *in vivo *study that *H. influenzae *can persist in a human bronchiolar xenograft for at least three weeks [5]. This suggests that the organism can survive and persist in protected biological compartment(s).

The ability of *H. influenzae *to survive antibiotic treatment and reappear when growth is favorable may be responsible for the reseeding of the middle ear observed in chronic otitis media. Often, middle ear fluid from children presenting with otitis media with effusion are sterile when cultured, but PCR analysis of the fluid has determined the presence of *H. influenzae *[6]. Further, RT-PCR studies of this sterile fluid have shown the presence of bacterial mRNA, confirming that the bacteria are alive and persisting in a viable but nonreplicative state [7].

Persistence was investigated *in vitro *using a NTHi strain that was susceptible to β-lactam antibiotics. This strain was allowed to invade a human respiratory epithelial cell monolayer for 24 hours, which was subsequently treated with a 4 hour incubation in 10 × MIC concentrations of the β-lactam antibiotics ampicillin, imipenem, cefuroxim, amoxycillin/clavulanic acid, or cephalothin. The antibiotics killed all the extracellular bacteria, but none of the intra- or paracellular bacteria, suggesting that the organism was not replicating inside or between the epithelial cells [8]. Non-replicating bacteria are not susceptible to the cidal action of β-lactam and aminoglycoside antibiotics.

During a study aimed at identifying genes associated with virulence in pathogenic strains of the Gram-negative, strict anaerobe *Dichelobacter nodosus*, the causative agent of ovine footrot, Katz *et al*. [9] reported the discovery of a novel area of the chromosome that hybridized to all virulent strains tested, but to only 23% of the avirulent strains studied. They designated the four genes found on this fragment as *vapA*-*D*, for ***v***irulence-***a***ssociated ***p***roteins. Homologues of these genes appear on the chromosomes and plasmids of a number of pathogenic microorganisms, including *Neisseria gonorrhoeae*, *Helicobacter pylori, Reimerella anatipestifer *and *Actinobacillus actinomycetemcomitans*. The chromosome of *H. influenzae *strain Rd KW20 (hereinafter referred to as strain Rd) contains *vapA*, *vapBC*, and *vapD *homologues, with one pair, *vapBC*, in duplicate. The genome organization of the *vap *genes in *H. influenzae *differs from that of *D. nodosus*, in that *vapA*_*Hi *_(HI1250) is preceded by a conserved hypothetical protein, HI1251, and both genes are likely transcribed as an operon. As well, *vapD*_*Hi *_is flanked by a gene encoding another conserved hypothetical protein, HI0451 (which we have named *vapX*_*Hi*_), again in an apparent operon configuration (8 nucleotides separate HI0450 and HI0451). To determine if the *vap *homologues played a role in the persistence of NTHi, we chose *vapD*_*Hi *_(HI0450) for further study, since this protein was found in a proteomic survey to be expressed in the soluble fraction of strain Rd [10]. VapD_*Hi *_is 40% identical and 67% similar to the *Dichelobacter *VapD and belongs to the Cluster of Orthologous Groups (COG) 3309 and Pfam 04605, termed the "N-terminal conserved domain of VapD".

## Results

### Mutation of *vapD*_*Hi *_results in attenuated survival in human endothelial cells

When the Rd *vapD*_*Hi *_mutant strain was used to invade the *in vitro *model of the blood-brain barrier, human brain microvascular endothelial cells (HBMEC) in 12-well plates, the amount of gentamicin-resistant bacteria recovered from the monolayer after three hours declined to approximately 60% of wild type levels, an average of 2.2 × 10^3 ^CFU/ml for the wild-type strain Rd versus 1.3 × 10^3 ^CFU/ml for the Rd *vapD*_*Hi *_mutant (n = 3 (number of independent assays performed in at least duplicate); P < 0.05, Student's *t *test). No significant difference was observed between the wild type and *vapD*_*Hi *_mutant in adherence to the human cell monolayers: the average number of cell-associated bacteria (both adherent and invaded) recovered for strain Rd was 1.5 × 10^5 ^CFU/ml and 1.8 × 10^5 ^CFU/ml for the Rd *vapD*_*Hi *_mutant (n = 3; P > 0.05).

To determine if the phenotype of attenuated survival observed in the Rd *vapD*_*Hi *_mutant was a general phenomenon and not restricted to strain Rd, another isogenic pair was constructed and analyzed using a different strain, R3001. R3001 is a bronchoalveolar lavage isolate from a pediatric cystic fibrosis patient, and is considered invasive since it came from a normally sterile site [5]. The average number of gentamicin-resistant bacteria recovered from HBMEC monolayers was 1.2 × 10^5 ^CFU/ml for the parent strain R3001 versus 7.1 × 10^4 ^CFU/ml for the R3001 *vapD*_*Hi *_mutant (n = 3; P < 0.05). Although the absolute numbers of bacteria recovered were higher with strain R3001 than with Rd (as is often observed with invasive isolates), the attenuation of survival inside the HBMEC monolayer of ≤ 60% observed in the strain with a *vapD*_*Hi *_mutation was maintained. There was no significant difference between the wild-type R3001 and the R3001 *vapD*_*Hi *_mutant in adherence to the monolayer: the average numbers of cell-associated bacteria recovered for strain R3001 were 1.7 × 10^7 ^CFU/ml versus 1.3 × 10^7 ^CFU/ml for R3001 *vapD*_*Hi *_(n = 4; P > 0.05). Unlike Rd, strain R3001 carries the high molecular weight (HMW) adhesins, which may account for its more efficient adherence to the HBMEC monolayer [11]. No significant difference in the growth rates of either of the *vapD*_*Hi *_mutants versus their cognate parent strains were observed, whether grown in bacteriological media (sBHI broth) or on HBMEC or NCI-H292 monolayers (data not shown).

### Mutation of *vapD*_*Hi *_results in diminished long-term survival inside human respiratory epithelial cells

To determine if the *vapD*_*Hi *_mutation would affect the ability of *H. influenzae *to survive inside human respiratory epithelial cells over a longer period of time, 18-hour invasion assays were performed using NCI-H292 cells. The number of gentamicin-resistant bacteria recovered from the NCI-H292 monolayer after 18 hours for the parent strain Rd was an average of 6.4 × 10^4 ^CFU/ml versus 3.2 × 10^4 ^CFU/ml for the Rd *vapD*_*Hi *_mutant (n = 3; P < 0.05). This represents a 50% reduction in survival of the *vapD*_*Hi *_mutant within epithelial cells as compared to the parent strain, more attenuation than was seen for the three hour assays.

### Transcomplementation of Rd *vapD*_*Hi*_

The *vapD*_*Hi *_locus from strain R3001 was cloned into the mobilizable broad host range plasmid pDD515, creating pDD564, and conjugally transferred into the Rd *vapD*_*Hi *_mutant (Table 1). The plasmid pDD515 is a derivative of the IncQ plasmid RSF1010 and has an approximate copy number of 12 per chromosome in *H. influenzae *[12]. The survival inside HBMEC of strain Rd (pDD515) was within 5% of strain Rd without the vector in identical assays (data not shown). Carrying a *vapD*_*Hi *_locus *in trans *restored wild-type survival of Rd *vapD*_*Hi *_(pDD564) within HBMEC monolayers. The amount of gentamicin-resistant bacteria recovered from the endothelial cell monolayer after a three hour invasion assay was an average of 8.0 × 10^2 ^CFU/ml for Rd (pDD515), the vector control, and 7.6 × 10^2 ^CFU/ml for the mutant strain that carried the wild-type *vapD*_*Hi *_allele *in trans*, Rd *vapD*_*Hi *_(pDD564) (n = 3; P > 0.05), indicating that there was no significant difference in the survival inside HBMEC monolayers of the wild type strain versus the transcomplemented strain. These data confirm that the phenotype of attenuated survival was due to the interruption in *vapD*_*Hi *_and not to polar effects.

The R3001 *vapD*_*Hi *_mutant mirrored the survival defect seen with the Rd *vapD*_*Hi *_mutant, at approximately 60% of wild-type R3001 levels. However, attempts to conjugate the mobilizable broad host range plasmid carrying the *vapD*_*Hi *_locus, pDD564, into strain R3001 for transcomplementation studies failed repeatedly. This clinical isolate likely has a plasmid or an origin of replication of the same incompatibility group incorporated into its chromosome and therefore will not maintain the broad host range plasmid for transcomplementation [13].

### Reverse-transcriptase PCR of bacteria from human cell monolayers

**Figure 1:**
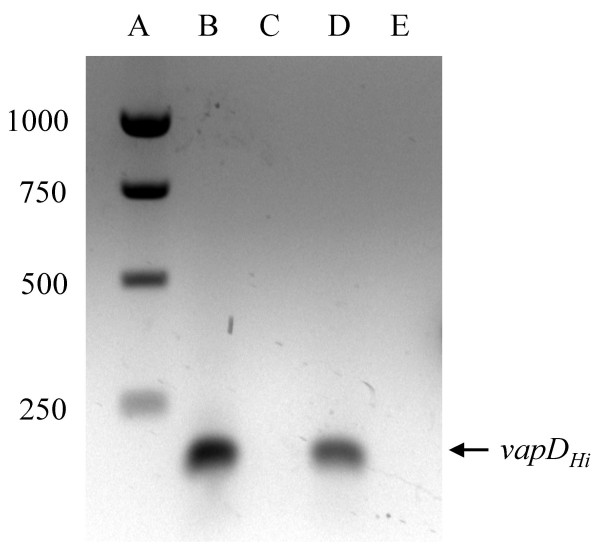
**Agarose gel of RT-PCR products. **Reverse-transcriptase PCR (RT-PCR) of total RNA isolated from wild-type strain Rd after 18 hours of contact with either HBMEC (human endothelial cell) or NCI-H292 (human epithelial cell) monolayers. Lane A: Molecular weight marker (Promega 1 Kb ladder); lane B: RNA from Rd on HBMEC monolayers; lane C: Negative control for lane B (no reverse transcriptase added prior to PCR); lane D: RNA from Rd on NCI-H292 monolayers; lane E: Negative control for lane D (no reverse transcriptase added prior to PCR).

To determine if the *vapD*_*Hi *_locus was transcribed during contact with a human cell monolayer, total RNA was isolated from the wild-type strain Rd recovered after 18 hours on HBMEC or NCI-H292 monolayers and was used as the template for RT-PCR. Figure 1 shows the 153 bp band amplified with the *vapD*_*Hi*_-specific primers, with a molecular weight marker in lane A. The template for lane B is Rd RNA from HBMEC endothelial cell monolayers, lane C is its cognate negative control, with no reverse transcriptase added to the RNA prior to PCR amplification. Lane D shows the results using Rd RNA from NCI-H292 epithelial cell monolayers; lane E is its cognate negative control. The *vapD*_*Hi*_locus is transcribed when strain Rd is in contact with either human epithelial or endothelial cell monolayers.

### PCR survey of *vapD*_*Hi*_

In order to estimate the carriage rate of *vapD*_*Hi *_among the highly heterologous NTHi strains, a PCR survey of 59 commensal and disease-associated strains was undertaken (Table 2). The *vap*HI primer set was used. In Rd, these primers amplify a 769 bp PCR product that includes the full-length *vapD*_*Hi *_gene.

Purified chromosomal DNA preparations from 53 randomly-chosen NTHi strains and one each of the six capsular serotypes of *H. influenzae *(types a through f) from the American Type Culture Collection (ATCC) reference strains described in Table 2 were subjected to PCR with the *vap*HI forward and reverse primers. The NTHi strains included nasopharyngeal, blood, CSF, middle ear, tracheal aspirate, and sputum isolates. A PCR product was amplified in 100% of the strains. All of the ATCC encapsulated reference strains and Rd displayed a full-length *vapD*_*Hi*_allele. Ninety-three percent of the nasopharyngeal strains carried a full-length allele, as did 71% of the blood and CSF isolates, and 50% of the middle ear, tracheal aspirate, and sputum isolates. Overall, only ten strains of the 59 included in the study displayed a truncated gene.

### Sequencing of the truncated *vapD*_*Hi *_allele

**Figure 2:**
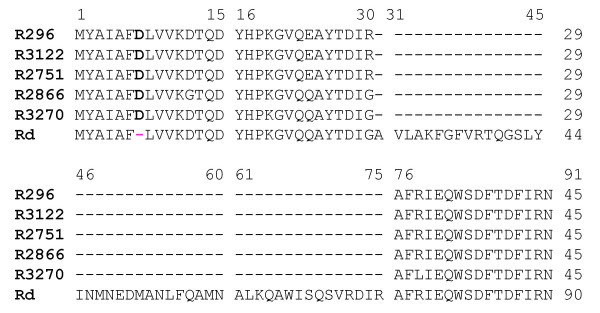
**Multiple sequence alignment of truncated VapD_*Hi *_proteins. **Wild type full-length VapD_*Hi *_protein sequence from strain Rd is included for comparison. R296: middle ear isolate; R3122: blood isolate; R2751: sputum isolate; R2866: blood isolate; R3270: nasopharynx isolate.

To study the truncated *vapD*_*Hi *_in more detail, five out of the ten alleles that represented the smaller isoform of *vapD*_*Hi *_from the PCR survey were sequenced on both strands. It was found that, in all cases, the gene had undergone a deletion event that had left the protein in frame, but missing 46 amino acids from the interior of the protein, resulting in a 45 amino acid protein rather than the full-length 91 amino acid protein (Figure 2). This corresponds to the loss of Rd genome coordinates 473123 to 473263. In addition, all of the smaller alleles had an aspartate residue inserted at position #7 as compared to Rd, which has a leucine at that position. The significance of this is unclear, as the full-length R3001 *vapD*_*Hi *_allele, which did transcomplement the Rd *vapD*_*Hi *_mutant, also has an aspartic acid inserted at the same position, resulting in a 91 amino acid protein. Interestingly, the VapD homologues from *N. gonorrhoeae*, *H. pylori *and *A. actinomycetemcomitans*, as well as VapD in *D. nodosus*, all have an aspartate at that position. Rd appears to be the only *H. influenzae *strain studied which lacks that particular residue.

### Full-length VapD_*Hi *_homodimerizes

Using an *E. coli*-based protein-protein interaction system that is dependent upon the DNA-binding domain (DBD) of LexA, homodimerization of identical protein subunits can be quantitated [14,15]. In this system, protein-protein interactions result in a LexA dimer that is active as a repressor, and consequently, the beta-galactosidase activity of the reporter strain (SU101) diminishes. Full-length VapD_*Hi *_from strain R3001 was ligated to the DBD of LexA in plasmid pDD559 and the clones were analyzed on MacConkey agar with lactose. If there was no homodimerization of the LexA::VapD_*Hi *_fusion protein, the colonies appeared red on MacConkey agar, as the native level of beta-galactosidase expression in the reporter strain was not inhibited. If the subunits interacted, the colonies appeared pale on MacConkey, as the engineered LexA operator controlling the *lacZ *reporter gene had been repressed by a homodimer of the LexA fusion protein. This repression was quantitated by beta-galactosidase activity assays. Each measurement is the mean of at least three experiments performed in triplicate.

It was found that VapD_*Hi *_interacted strongly with itself. The beta-galactosidase activity of the reporter strain SU101 carrying the vector control (pSR658) was an average of 975 (± 29) Miller units, and the activity of SU101 with the LexA::VapD_*Hi *_fusion (pDD559) was an average of 16 (± 1) Miller units, indicating strong interaction. Full-length VapD_*Hi *_forms homodimers *in vivo*. This protein may also form higher-order multimers, since this would result in a number of dimeric forms being available to act as a repressor of *lacZ *transcription in the reporter strain.

### Truncated VapD_*Hi *_does not homodimerize, but interacts with full-length VapD_*Hi*_

Homodimerization assays with the small allele revealed that the subunits of the truncated VapD_*Hi *_did not interact efficiently. The vector control for the homodimerization assays SU101 (pSR658) yielded 1490 (± 31) Miller units, and SU101 carrying the LexA fusion to the truncated VapD_*Hi *_protein from strain R2866 (pDD577) displayed 1357 (± 54) Miller units of beta-galactosidase activity, showing little repression in this system.

Since the wild-type VapD_*Hi *_subunits homodimerized strongly but the truncated subunits did not, the truncated subunit and the full-length subunit were examined for interaction in heterodimerization assays. In the reporter strain SU202, a LexA operator with a mutated half-site was engineered upstream of the *lacZ *gene. This strain was then transformed with two compatible plasmids, one that carried a fusion of the truncated VapD_*Hi *_with a wild-type LexA DBD, and one that carried the full-length VapD_*Hi *_fusion to a mutated LexA DBD that only recognized the mutated LexA operator half-site in SU202 [15]. If a heterodimer of a truncated subunit and a full-length subunit was formed, a functional LexA repressor could recognize the hybrid operator and repress transcription of *lacZ*. It was determined that the truncated and full-length subunits could interact with each other. The vector control for the heterodimerization assays (SU202 with pSR658 and pSR659) yielded 1855 ± 196 Miller units, and SU202 carrying the LexA fusion to the truncated VapD_*Hi *_protein from strain R2866, pDD577, coupled with the mutated LexA DBD fusion to the full-length VapD_*Hi *_protein from strain R3001, pDD561, resulted in 792 ± 19 Miller units of beta-galactosidase activity, showing that the truncated subunit did heterodimerize with the full-length VapD_*Hi *_subunit.

### Truncated VapD_*Hi *_does not transcomplement the mutant and has a dominant-negative effect in the wild-type strain

To investigate whether the truncated VapD_*Hi *_protein could transcomplement a mutation in the full-length gene, the truncated locus from strain R2866 was cloned into the mobilizable broad host range vector pDD515, creating pDD594, and conjugally transferred into the Rd *vapD*_*Hi *_mutant strain. Three-hour survival assays using HBMEC monolayers were performed, and the number of gentamicin-resistant bacteria recovered from the monolayer was an average of 5.6 × 10^2 ^CFU/ml for Rd (pDD515), the vector control, versus 1.9 × 10^2 ^CFU/ml for Rd *vapD*_*Hi*_(pDD594) (n = 3; P < 0.005). Survival in HBMEC by the mutant strain did not increase to wild-type levels, as was the case with the Rd *vapD*_*Hi *_mutant transcomplemented with a full-length *vapD*_*Hi *_allele on pDD564.

Since subunits of the full-length VapD_*Hi *_and the truncated protein interacted, the plasmid pDD594 carrying the truncated allele was conjugally transferred into wild-type strain Rd to determine whether expression of the small protein would interfere with the function of the wild-type VapD_*Hi *_protein. The strain was used in three-hour assays of HBMEC monolayers, and it was found that Rd (pDD594) was attenuated in human cell survival as compared to Rd (pDD515), the wild-type strain carrying the vector without an insert. The average number of gentamicin-resistant bacteria recovered from the monolayer for Rd (pDD515), the vector control, was 5.7 × 10^2 ^CFU/ml versus 1.8 × 10^2 ^CFU/ml for Rd (pDD594) (n = 3; P < 0.005). The *in trans *expression of a truncated *vapD*_*Hi *_allele in the wild-type strain Rd resulted in a dominant-negative effect on survival within HBMEC monolayers.

### Expression of *vapD*_*Hi *_in *Escherichia coli *DH5α results in cell growth arrest

To test the hypothesis that VapD_*Hi *_constituted the toxin, and that VapX_*Hi *_encoded the antitoxin portion of a TA locus, both proteins were expressed in an *E. coli *background. *E. coli *does not contain a homologue of either protein. Initially, the *vapD*_*Hi *_gene, HI0450, was cloned into the pTrcHisA vector (Invitrogen, Carlsbad, CA). This resulted in *vapD*_*Hi *_being under the control of the strong P_*trc *_promoter, which is repressed for the most part until induced by IPTG. This plasmid was designated pDD560. Both the vector control, DH5α (pTrcHisA), and DH5α (pDD560) were grown to mid-log phase in LB broth with 100 μg/ml ampicillin and aliquots were spread on LB agar plates with 100 μg/ml ampicillin and 0.1 mM IPTG. Strain DH5α (pTrcHisA) grew on the plates, but DH5α (pDD560) did not, indicating that induction and overexpression of *vapD*_*Hi *_was toxic to *E. coli*. The putative antitoxin VapX_*Hi*_, was then cloned into the pTrcHisA vector. No growth disruption occurred in DH5α with the overexpression of VapX_*Hi *_alone. The *vapX*_*Hi *_gene plus the *lacI*^*q *^gene were then subcloned into the broad host range mobilizable plasmid, pDD515, resulting in pDD672. This strategy allowed the DH5α test strain to carry two compatible plasmids, one which encoded the *vapD*_*Hi *_gene (pDD560) and the other expressing the *vapX*_*Hi *_antitoxin gene (pDD672). Both genes were under the control of a P_*trc *_promoter and were therefore both repressed until induced by IPTG. When both genes were induced with 0.1 mM IPTG on LB agar plates that contained 100 μg/ml ampicillin and 10 μg/ml chloramphenicol in strain DH5α (pDD560, pDD672), growth was restored. This indicated that the concurrent expression of the *vapX*_*Hi *_antitoxin with the *vapD*_*Hi *_toxin ameliorated the cell growth arrest observed with expression of *vapD*_*Hi *_alone, and that *vapX*_*Hi *_was necessary for this rescue.

## Discussion

Mutation of the *vapD*_*Hi *_allele in strains Rd and R3001 resulted in attenuation of survival within both HBMEC and NCI-H292 monolayers, suggesting that in *H. influenzae*, the presence of a functional VapD_*Hi *_facilitates persistence within epithelial and endothelial cells. Mutants invaded and survived in human cells at ≤ 60% of wild-type levels. Although relatively modest, this level of attenuation has been observed during the mutational analysis of other *Haemophilus *virulence factors, such as opacity-associated protein A as well as the high molecular weight (HMW) proteins [11,16]. *H. influenzae *survival within human cells is multifactorial, and our data indicate that VapD_*Hi *_contributes to this process. However, strain Rd contains three other *vap *genes (*vapA*_*Hi*_, *vapB*_*Hi*_, and *vapC*_*Hi*_), and it is possible that these *Haemophilus vap *genes act synergistically, such that multiple mutations may result in a more attenuated survival phenotype. Indeed, a recent study has determined that a chromosomally-located homologue of the VapBC locus acts as a toxin-antitoxin module in the spirochete *Leptospira interrogans *[17]. It would be interesting to characterize a *Haemophilus *strain with mutations in all the *vap *genes.

Neither of the *vapD*_*Hi *_mutants displayed differences in adherence to the monolayers compared to the parent strain, so the defect occurred after binding and affected the organism's ability to persist inside or between cells. Interestingly, the *vapD*_*Hi *_mutants were not attenuated in growth rate when compared to the parent strains, either in bacteriological media or on the surface of human cell monolayers. The observed survival attenuation of the mutants could be transcomplemented with a full-length allele from a clinical isolate, R3001, demonstrating that the phenotype was due to the mutation in *vapD*_*Hi *_and not polar effects. A truncated allele from another clinical isolate, R2866, did not transcomplement the Rd *vapD*_*Hi *_strain, indicating that the truncated protein was not functional *in vivo*. RT-PCR analysis confirmed that the full-length *vapD*_*Hi *_locus in Rd was transcribed during contact with both epithelial and endothelial cells. VapD_*Hi *_has also been identified in the soluble fraction of strain Rd grown in bacteriological media [10]. It remains to be seen if the transcription of this locus increases upon contact with human cells.

Results of a PCR survey on 59 randomly-chosen strains showed that nearly all of the genetically highly heterologous NTHi commensal isolates surveyed (93%) carried a full-length *vapD*_*Hi *_allele on their chromosomes, suggesting that maintenance of a functional *vapD*_*Hi *_gene was beneficial to survival in this niche (Table 2). Of the invasive strains isolated from the blood or cerebrospinal fluid, 71% retained a full-length allele. Fifty percent of the isolates from sputum, tracheal aspirates, and the middle ear carried the full-length allele. Finally, all the encapsulated strains tested contained a full-length allele. It must be noted, however, that this analysis was not an exhaustive study, since a limited number of strains were included. Many clinical NTHi strains have previously been shown to express various virulence factors that enhance adherence and invasion into human cells which are not found in the sequenced Rd strain [11,18-20]. The strains identified in this PCR study that lack a functional *vapD*_*Hi *_allele probably compensate for its loss with genes that are not found in Rd, and these "extra genes" may well include other TA loci.

The calculated molecular mass of the VapD_*Hi *_protein in Rd is approximately 10 kilodaltons, and small bacterial proteins often form multimers. Full-length VapD_*Hi *_subunits exhibited strong homodimerization in a LexA-based protein-protein interaction system, and this may indicate that the subunits form higher-order multimers such as homotrimers or homotetramers *in vivo*. However, the protein encoded by the truncated allele did not homodimerize in the same system, further evidence of its loss of function. Interestingly, the truncated subunit did interact with full-length subunits in heterodimerization assays. Further evidence of this heterodimerization *in vivo *was that the expression of the truncated subunit in the wild-type strain resulted in a dominant-negative effect on survival within HBMEC monolayers, the levels of which mimicked the attenuation observed with the *vapD*_*Hi *_mutation. This was likely due to truncated subunits forming hybrid complexes with full-length subunits and interfering with their structure and/or function, resulting in the observed dominant-negative phenotype.

The activities of only a few toxins encoded by TA loci have been elucidated thus far. Two specific targets of plasmid-encoded toxins have been identified: CcdB of the F plasmid and ParE of plasmid RK2 inhibit DNA gyrase, and Kid of plasmid R1 was previously thought to interact with DnaB helicase, but has recently been shown to cleave cellular mRNA [21-23]. The target and function of a toxin from a chromosomally-encoded TA locus (*relBE*) was determined to be cleavage of mRNA in the ribosomal A site [24]. Strain Rd contains *relBE *homologues (HI0710 and HI0711) as well as a homologue of *higA *(*h*ost *i*nhibition of *g*rowth antidote protein) from plasmid Rts1. The *higBA *TA locus is unusual in that the toxin gene (*higB*) exists upstream of the antidote protein (*higA*). Interestingly, VapA_*Hi *_of strain Rd is 29% identical and 53% similar to HigA.

While the data acquired in this study suggests that VapD_*Hi *_and VapX_*Hi *_form a toxin-antitoxin pair, it is unusual to find homologues of VapX_*Hi *_only in *H. influenzae *and the gonococcal plasmid. *H. influenzae *strains R2846 and R2866 both have a truncated *vapD*_*Hi *_toxin gene, and possess a *vapX*_*Hi *_which is 100% identical to that gene in strain Rd. Their respective genomes can be searched at . Interestingly, there are complete genome sequences available for two isolates of *H. pylori, N. meningitidis *and *Haemophilus somnus*, but only one strain of each has a homologue of *vapD*_*Hi*_. Thus, several features of the *vapD*_*Hi*_/*vapX*_*Hi *_gene pair are unusual for a toxin-antitoxin locus.

## Conclusions

Persistence of NTHi is important in the progression of disease caused by this organism. Many investigators have previously reported the discovery of a number of virulence factors associated with adherence, invasion and survival of NTHi inside human cells [1,3,4,16,18,25]. Here we report a locus that is also involved in the pathogenesis of nontypeable *H. influenzae*. Further studies are required to fully characterize the mechanism of VapD_*Hi *_function and to define its role in the modulation of NTHi persistence in human cells.

## Methods

### Bacterial strains, media and reagents

All *H. influenzae *strains used are listed in Table 2. *H. influenzae *was grown on chocolate agar (36 g Difco GC medium, 10 g hemoglobin, 10 ml Difco Supplement B (Becton Dickinson, Sparks, MD), 5,000 Units bacitracin per liter) or supplemented BHI (sBHI) broth or agar (37 g brain heart infusion media ± 15 g Bacto agar per liter (Remel, Lenexa, KS) with 10 μg/ml β-NAD, 10 μg/ml heme-histidine, and 5 Units/ml bacitracin). Strains containing the TSTE cassette [26] were grown on media with 15 μg/ml ribostamycin sulfate (CalBioChem, San Diego, CA). Bacteria were diluted for plating with PBS-G (phosphate-buffered saline (pH 7.0) with 0.1% gelatin). *Escherichia coli *strains used were DH5α, to clone fragments of NTHi DNA; DD12, as the host strain in conjugations; and SU101 or SU202 as the reporter strains for the homodimerization and heterodimerization assays, respectively [14,15]. T4 bacteriophage was obtained from the American Type Culture Collection (ATCC #11303). Antibiotics and other chemicals were from Sigma-Aldrich (St. Louis, MO). Restriction enzymes, deoxyribonucleotides, T4 DNA polymerase, and T4 DNA ligase were from Promega (Madison, WI). Enzymes and other reagents for PCR were from Eppendorf Scientific (Westbury, NY) and Bioline (Canton, MA). Enzymes and reagents for reverse transcriptase PCR (MasterAmp RT-PCR Kit) were from Epicentre Technologies (Madison, WI). Oligonucleotide primers were synthesized by Integrated DNA Technologies (Coralville, IA). DNA sequencing was performed by the University of Missouri DNA Core Facility (Columbia, MO), Davis Sequencing, LLC (Davis, CA), and the DNA Core Facility at Seattle Biomedical Research Institute (Seattle, WA). Plasmids were isolated using the Wizard SV Plus Plasmid Miniprep kit, PCR products and restriction digests were purified using the Wizard PCR Prep kit, and total bacterial RNA was isolated using the SV Total RNA Isolation System (Promega, Madison, WI).

### Plasmids and conjugations

For transcomplementation, the 276 bp R3001 *vapD*_*Hi *_allele, along with 269 bp upstream and 227 bp downstream, was PCR-amplified using primers *vap*HI forward 5'-TATGTCTAGACAGTCGCTTCATAAGC-3' and *vap*HI reverse 5'-CCATTCTAGATTTGAGGTTAAATATGG-3'. Both primers included a *XbaI *site (underlined) and amplified Rd genome coordinates 472803 to 473572. The product was sequenced and cloned both into the *XbaI *site of pBluescript SK^+ ^(creating plasmid pDD562) and into the *NheI *site of pDD515, a mobilizable broad-host range vector that could be conjugally transferred into and stably maintained in *H. influenzae *[12], creating plasmid pDD564 (Table 1). Plasmid pDD564 was used for transcomplementation of Rd *vapD*_*Hi*_. The same primers were used to PCR amplify the 135 bp truncated *vapD*_*Hi *_allele from strain R2866, the product of which was cloned into the *NheI *site of pDD515, creating pDD594. The insert was sequenced, then the plasmid was conjugally transferred into both Rd *vapD*_*Hi *_and wild-type Rd. Conjugations were carried out as previously described [12]. For allelic exchange, the plasmid pDD563 was constructed, which consisted of pDD562 with an interruption of *vapD*_*Hi *_by an aminoglycoside phosphotransferase gene (*aph (3')II*). Specifically, the 2184 bp *BamHI *fragment from pTSTE [26], which had been rendered blunt-ended with mung bean nuclease, was inserted into the *BsaBI *site of *vapD*_*Hi*_. For the homodimerization assays, the plasmids pDD559 and pDD577 were derived from pSR658 and carried the NTHi strain R3001 *vapD*_*Hi *_allele or the NTHi strain R2866 *vapD*_*Hi*_allele fused in-frame to the wild-type LexA DNA-binding domain, respectively [27]. For the heterodimerization assays, the plasmid pDD561 derived from pSR659 was constructed, which consisted of the R3001 *vapD*_*Hi *_allele fused in-frame to the mutated LexA DNA-binding domain.

### Mutation of *vapD*_*Hi*_

The *vapD*_*Hi *_genes in Rd and strain R3001 were disrupted by allelic exchange. Briefly, strains Rd and R3001 were made competent using the M-IV media technique [28] and pDD563 linearized with *XmnI *was used to transform each strain. Transformants were selected on chocolate agar supplemented with 15 μg/ml ribostamycin sulfate (CalBioChem, San Diego, CA). The insertion in *vapD*_*Hi *_was confirmed by Southern blotting using a digoxygenin-labeled denatured PCR fragment of *vapD*_*Hi *_as the probe. The orientation of the aminoglycoside phosphotransferase cassette was determined by PCR using a primer that originated inside the *aph (3')II *gene and another that flanked *vapD*_*Hi*_. The resistance gene was found to be transcribed in the opposite orientation of *vapD*_*Hi *_in both strains.

### Cell culture

Human brain microvascular endothelial cells (HBMECs) were a gift from K. S. Kim [29]. Cells were passaged in collagen-1 coated T-25 flasks and monolayers for invasion assays were grown in 12-well collagen-1 coated BioCoat plates (Becton Dickinson, Bedford, MA). HBMEC media contained 760 ml RPMI 1640 with 25 mM HEPES and 2 mM L-glutamine, 100 ml heat-inactivated fetal calf serum, 10 ml each of 200 mM L-glutamine, 100× MEM non-essential amino acid solution, 100× MEM vitamin solution, 100 mM MEM sodium pyruvate solution (Gibco, Grand Island, NY), and 100 ml heat-inactivated NuSerum V (Becton Dickinson, Bedford, MA) per liter. Media was changed every two days and cells were passaged every 3–5 days. Monolayers were seeded at a density of ~2.0 × 10^5 ^cells per well and used 48 to 72 hours after seeding.

NCI-H292 human respiratory epithelial cells (ATCC catalogue # CRL-1848) were passaged in collagen-1 coated T-25 flasks and monolayers for invasion assays were grown to confluency in 12-well collagen-1 coated BioCoat plates (Becton Dickinson, Bedford, MA). NCI-H292 media consisted of 870 ml RPMI 1640 medium with 25 mM HEPES and 2 mM L-glutamine, 10 ml of 100 mM MEM sodium pyruvate solution, 10 ml of 7.5% w/v sodium bicarbonate solution (Gibco, Grand Island, NY), 10 ml of 450 mg/ml filter-sterilized glucose solution, and 100 ml heat-inactivated fetal calf serum per liter. As above, media was changed every two days and cells were passaged every 3–5 days. Monolayers were seeded at a density of ~2.5 × 10^5 ^cells per well and used 72 to 96 hours after seeding.

### Invasion and survival assays

Gentamicin-resistance invasion and survival assays were performed on HBMEC and NCI-H292 monolayers as previously described [5]. Briefly, the inoculum used was 1.0 – 5.0 × 10^6 ^CFU of *H. influenzae *in a volume of 1 ml per well of a 12-well plate (an MOI of ≤ 10:1). After a 3 or 18 hour incubation in an atmosphere of 5% CO_2 _at 37°C, each monolayer was extensively washed with Dulbecco's PBS and 1.5 ml of media containing 100 μg/ml gentamicin was added to each well. Following a subsequent one hour incubation in the antibiotic, the wells were again washed extensively, harvested with 1% saponin, diluted in PBS-G and plated on chocolate or sBHI agar for viable intracellular CFU/ml. To quantitate total cell-associated bacteria (both intracellular and adherent), wells were also harvested and plated after the first wash and prior to gentamicin addition.

### Methods for statistical analysis

Statistical analyses were performed using the statistical analysis functions of Microsoft Excel (Microsoft Office 1997). For most comparisons of data, the Student's *t*-test was used and *P*-values of <0.05 were considered to indicate statistically significant differences.

### Reverse transcriptase PCR (RT-PCR)

Total RNA was isolated using the SV Total RNA isolation system (Promega, Madison, WI) from the wild type strain Rd recovered from the media of 18-hour invasions of either HBMEC (endothelial) or NCI-H292 (epithelial) monolayers. Standard procedures were used, with the modification that two separate DNAseI incubations were performed instead of the single one recommended with the kit. RT-PCR using the MasterAmp RT-PCR kit was then performed as per the manufacturer's instructions (Epicentre Technologies, Madison, WI). Negative controls of no reverse transcriptase added to the RNA followed by traditional PCR using Biolase DNA polymerase (Bioline, Madison, WI) were used to ensure that both RNA preparations were free of contaminating DNA. The primers for RT-PCR, 450 RT for (5'-CAGGCTTATACAGACATTGG-3') and 450 RT rev (5'-TCGTACCGACTGAGAAATCC-3') amplified a 153 bp internal portion of the *vapD*_*Hi *_cDNA.

### Protein-protein interaction assays

The *vapD*_*Hi *_alleles from strains R3001 (full-length) and R2866 (truncated) were amplified by PCR and fused in-frame to the LexA DNA-binding domain (DBD) in pSR658, resulting in pDD559 and pDD577, respectively, and used to transform the reporter strain SU101 for homodimerization assays [27]. Briefly, strain SU101 carries a *lacZ *gene controlled by a wild-type LexA operator site [14]. If a homodimer of two LexA DBD fusions was formed, the complex could bind to the LexA operator region and shut down transcription of *lacZ*, resulting in diminished levels of beta-galactosidase. The *vapD*_*Hi *_allele from strain R3001 was also fused in-frame to the mutated LexA DNA-binding domain in pSR659, creating pDD561. The compatible plasmids pDD561 (full length *vapD*_*Hi*_) and pDD577 (truncated *vapD*_*Hi*_) were both used to transform the reporter strain SU202 for heterodimerization assays. Strain SU202 [14] also has a *lacZ *gene controlled by a LexA operator, but this operator site is engineered such that only a mutated LexA DBD subunit (coded on pSR659) can bind to one half-site, while a wild-type LexA DBD subunit (coded on pSR658) can bind to the other half-site. Consequently, only a heterodimer composed of one mutated LexA DBD fusion subunit and one wild-type LexA DBD fusion subunit could bind to this engineered site and decrease transcription of *lacZ *in SU202. Three independent beta-galactosidase assays were carried out in triplicate as previously described [27].

## Author's contributions

DAD conceived of the study, carried out the protein-protein interaction and molecular genetics work, and drafted the manuscript. JJ carried out the intracellular survival experiments. ALS supported the study and participated in its design and coordination. All authors read and approved the final manuscript.

## Figures and Tables

**Table 1:** Plasmids and *E. coli *strains used in this study.

**Plasmid**	**Description**	**Reference**
pBluescript	Cloning vector, Ap^R^	Stratagene
pDD515	RSF1010 *ori*; a mobilizable broad host range vector, Cm^R^	12
pDD559	pSR658 with *vapD*_*Hi *_in frame with wild-type LexA DNA-binding domain (DBD)	This work
pDD560	pTrcHisA with *vapD*_*Hi*_	This work
pDD561	pSR659 with *vapD*_*Hi *_in frame with mutant LexA DBD	This work
pDD562	pBluescript with *vapD*_*Hi *_region	This work
pDD563	pDD652 with an *aph(3')II *gene interrupting *vapD*_*Hi*_	This work
pDD564	pDD515 with a full-length *vapD*_*Hi *_region for transcomplementation	This work
pDD577	pSR658 with a truncated *vapD*_*Hi *_in frame with the wild-type LexA DBD	This work
pDD594	pDD515 with a truncated *vapD*_*Hi *_region for transcomplementation	This work
pDD672	pDD515 with the *vapX*_*Hi *_antitoxin for rescue	This work
pSR658	Wild-type LexA DBD fusion vector, Tc^R^	15
pSR659	Mutant LexA DBD fusion vector, Ap^R^	15
pTrcHisA	Cloning vector, Ap^R^	Invitrogen

**Strain**	**Description**	**Source**

DD12	Conjugal host strain	12
DH5α	Cloning strain	Promega
SU101	Homodimerization reporter strain	14
SU202	Heterodimerization reporter strain	14

**Table 2:** *H. influenzae *strains used in this study.

Strain*	**Description**	**Type**	***vapD***_***Hi***_**allele**^**†**^	**Prevalence**
R538	ATCC #9795	b	WT	100%
R539	ATCC #9006	a	WT	
R540	ATCC #9007	c	WT	
R541	ATCC #9008	d	WT	
R542	ATCC #8142	e	WT	
R543	ATCC #9833	f	WT	
R652, R3460*, R3539*, R3540*, R3541*, R3542*	Rd	NTHi	WT	

**Strain**	**Anatomical site**^**‡**^	**Type**	***vapD***_***Hi***_**allele**	**Prevalence**

C378, C483, C1591, C1607, R1624, R1625, R1627, R1632, R2754, R3254, R3256, R3258, R3259, R3262, R3264, R3265, R3266, R3267, R3268, R3269, R3271, R3273, R3274, R3276. R3277, R3282, R3283, R3285	Nasopharynx	NTHi	WT	93% (28/30)
R3257, R3270			TV	7%
R2752, R3001, R3027, R3157, R3543*	Sputum/Tracheal Aspirate/Ear	NTHi	WT	50% (4/8)
R296, R2751, R2846, R3151			TV	50%
C432, R228, R2777, R3168, R3252, R3278, R3279, R3280, R3330, R3331	Blood/CSF	NTHi	WT	71% (10/14)
R2866, R3122, R3164, R3169			TV	29%

*: Asterisk denotes derivative strains, i.e. R3460 is strain R652 carrying the vector pDD515. The marked strains are not included in computing the incidence of the wild-type *vapD*_*Hi *_allele versus the truncated allele. Independent strains are not marked with an asterisk. †: Type of *vapD*_*Hi *_allele: WT = wild type (91 amino acids); TV = truncated version (45 amino acids). ‡: Site from which strains were isolated.
